# The Crosstalk Between Metabolic Reprogramming and Histone Methylation in Tumor Immunity and Immunotherapy

**DOI:** 10.3390/cells15191786

**Published:** 2026-09-30

**Authors:** Jiayao Dou, Ruicheng Xue, Wei Gao, Fan Liang

**Affiliations:** 1 Department of Pathology and Pathophysiology, School of Basic Medicine, Shandong Second Medical University, Weifang 261000, China; 200454djy@stu.sdsmu.edu.cn; 2 Department of Medical Informatics, School of Basic Medicine, Shandong Second Medical University, Weifang 261000, China; 20250142@stu.sdsmu.edu.cn

**Keywords:** histone methylation, metabolic reprogramming, tumor immunometabolism, epigenetic regulation, tumor microenvironment

## Abstract

Histone methylation regulates chromatin structure and gene expression, whereas tumor metabolism provides bioenergetic and biosynthetic inputs. These processes are bidirectionally linked: S-adenosylmethionine supplies methyl groups for histone methyltransferases, α-ketoglutarate(α-KG) supports JmjC-domain demethylases, and D-2-hydroxyglutarate (D-2HG) can inhibit α-KG-dependent dioxygenases. Conversely, histone methylation can regulate metabolic gene expression, as illustrated by KMT2D-dependent enhancer remodeling and glycolytic gene expression in melanoma models. Lactate regulates chromatin through histone lactylation, a modification distinct from histone methylation; where the two intersect, as in the retinoblastoma H3K18la–SUZ12/PRC2–H3K27me3 circuit, the crosstalk is model-specific. This review examines these interactions in tumor cells and the immune microenvironment, emphasizing enzyme specificity, histone mark, cell type and experimental context. We highlight that changes in S-adenosylmethionine or α-KG do not produce uniform effects across enzymes, loci or cell states, and that tumor-associated macrophages are not captured by a fixed M1/M2 metabolic dichotomy. Mechanistic findings, preclinical results and early-phase clinical observations are considered separately; single-arm activity reported for valemetostat in adult T-cell leukemia/lymphoma is presented as activity in a defined cohort, not as an established survival benefit. Finally, we discuss how single-cell and spatial approaches can help resolve cell-type-specific and niche-specific interactions in heterogeneous tumors.

## 1. Introduction

Histone methylation is a conserved epigenetic modification that regulates chromatin structure and gene expression, thereby contributing to the maintenance of cellular identity and function. As an important post-translational modification (PTM), histone methylation mainly occurs on lysine and arginine residues of histone proteins, often within their N-terminal tails. The modification sites and the degree of methylation jointly regulate chromatin accessibility, thereby determining the biological effects of transcriptional activation or repression. As research on cancer has advanced, aberrant histone methylation patterns have been suggested to contribute to cancer initiation, progression, and therapeutic resistance. Histone methyltransferases (HMTs) and demethylases (HDMs) are responsible for “writing” and “erasing” methyl marks, respectively. The imbalance in their regulation disrupts the homeostasis of chromatin modifications, leading to the abnormal activation of oncogenes and silencing of tumor suppressor genes [1]. For example, histone H3.3 G34 mutations can alter H3K36 and H3K27 methylation *in cis*, whereas H3K27M mutations can impair PRC2-mediated H3K27 methylation in diffuse midline glioma; these effects are highly context dependent [2,3]. Additional sex combs-like (*ASXL*) proteins interact with chromatin-regulatory complexes, including PRC2 and Trithorax group (TrxG) complexes, and have been implicated in the regulation of histone modifications. Alterations in *ASXL*-family genes have been reported in acute myeloid leukemia (AML) and myelodysplastic syndrome (MDS) [4].

To meet the demands of rapid proliferation and survival under stress conditions, tumor cells undergo significant metabolic reprogramming. This metabolic plasticity is manifested in the abnormal activation of pathways such as enhanced glycolysis (Warburg effect), glutaminolysis and fatty acid oxidation, which not only supply energy for tumor cells but also generates metabolic intermediates that can act as substrates or cofactors for epigenetic enzymes, linking metabolic and epigenetic regulation [5,6,7]. Notably, metabolic intermediates such as S-adenosylmethionine (SAM), α-KG, lactate, and succinate can directly regulate the activity of chromatin-modifying enzymes, serving as key hubs connecting cellular metabolism and epigenetics [8,9,10]. For instance, α-KG-dependent dioxygenases mediate histone demethylation, while the oncometabolite 2-hydroxyglutarate (2-HG) produced by *IDH* enzyme mutations can inhibit the activity of these enzymes, triggering extensive epigenetic changes and promoting tumorigenesis [9,11]. Lactate, traditionally regarded as a metabolic waste product, has been shown to mediate histone lactylation, a novel epigenetic modification that regulates gene expression and immune evasion in the tumor microenvironment (TME) [12,13,14,15]. Histone lactylation is mechanistically distinct from histone methylation. However, the two pathways show regulatory crosstalk: lactate-driven lactylation can influence the expression of histone methylation-modifying enzymes such as *SUZ12*/PRC2, and both modifications can co-regulate chromatin accessibility at overlapping gene loci (see Section 2.2.4). A liver cancer study reported that metabolism and epigenetics form a bidirectional regulatory loop, revealing the core logic that metabolic reprogramming reshapes epigenetic modifications, and epigenetic modifications in turn regulate the expression of metabolic genes [16].

Recent studies have identified the crosstalk between histone methylation and tumor metabolism as an active area of cancer research with potential therapeutic implications. Epigenetic changes represented by aberrant histone methylation not only directly regulate the functions of metabolic enzymes and pathways, but also remodel the tumor immune microenvironment by modulating the expression of immune checkpoint molecules and the phenotype of immune cells [16,17,18]. For example, the epigenetic silencing of HLA class I molecules mediated by histone modifications and DNA methylation impairs tumor immune recognition, and epigenetic drugs targeting histone-modifying enzymes can effectively reverse this process [16]. Tumor-associated macrophages (TAMs) undergo metabolic and epigenetic reprogramming including changes in histone methylation, which affects their polarization direction and functions, thereby regulating tumor progression and immunotherapy response [17,18]. At present, combination strategies involving epigenetic modulators, metabolic interventions and immunotherapy are being investigated in preclinical models, although their efficacy remains context dependent [19,20,21].

Accordingly, this review focuses on the bidirectional relationship between histone methylation and tumor metabolism, with particular emphasis on tumor immunometabolism. Evidence from non-tumor systems is considered only when it clarifies a biochemical principle relevant to this axis, and is identified as indirect or hypothesis-generating. We distinguish mechanistic findings, preclinical results and early-phase clinical observations, and we do not interpret preclinical activity as established clinical benefit. Adjacent mechanisms, including histone lactylation, non-histone methylation, RNA-mediated regulation and autophagy, are discussed only when a defined molecular or metabolic link to histone methylation can be identified. Throughout, macrophage states are treated as heterogeneous and plastic rather than as fixed M1/M2 categories, and each mechanism is interpreted in relation to cell type, enzyme, genomic locus, metabolic state and experimental model. Clinical evidence, including tabulated findings, is presented with the relevant model and level of evidence specified.

## 2. The Dynamic Interplay Between Histone Methylation and Tumor Metabolism

### 2.1. Basic Mechanisms of Histone Methylation and Its Regulatory Role in Tumors

#### 2.1.1. Enzymatic Basis of Histone Methylation

Histone methylation is an important post-translational modification, and the dynamic balance of this process is primarily maintained by two classes of enzymes with antagonistic functions. Histone methyltransferases (HMTs) are responsible for transferring the methyl group provided by SAM to specific lysine or arginine residues on histone tails, while histone demethylases (HDMs) remove methyl marks, forming a reversible epigenetic regulatory loop [22]. Based on differences in substrate specificity and catalytic mechanisms, HMTs are mainly divided into two categories: histone lysine methyltransferases (KMTs) and protein arginine methyltransferases (PRMTs) (Table 1). Among them, KMTs include SET domain-containing proteins (e.g., *SUV39H1/2*, *G9a* and *EZH2*) and non-SET domain enzymes (e.g., *DOT1L*), which target key lysine residues of histone H3 (H3K4, H3K9, H3K27, H3K36 and H3K79) and H4 (H4K20), producing mono-, di-, or tri-methylation modification products, and different methylation states confer distinct transcriptional regulatory functions [23]. For example, H3K4me3 is enriched at transcription start sites (TSSs) and has been shown to regulate RNA polymerase II promoter-proximal pause-release rather than transcription initiation [24], while in selected cancer models, EZH2-containing PRC2 catalyzes H3K27me3 and contributes to transcriptional repression and malignant progression [25]. The catalytic activity of HMTs is tightly regulated by their domain assembly patterns and interactions with cofactors; for example, the COMPASS complex can regulate the level of H3K4 methylation by enhancing the enzymatic activity of *SET1* [26]. In contrast, HDMs are divided into the flavin adenine dinucleotide (FAD)-dependent LSD family (*LSD1/2*) and the Fe(II)- and α-KG-dependent JmjC domain family. These enzyme families differ in substrate and methylation-state preferences. Their activity and effects can also depend on cofactors, interacting proteins and cellular context [27,28].

Histone methylation can influence chromatin accessibility and the recruitment of methyl-mark readers, chromatin-remodeling complexes, and transcription factors. Its effects on gene expression depend on the specific mark and genomic context [61]. In addition, post-translational modifications of methyltransferases themselves (e.g., phosphorylation, acetylation, and ubiquitination) can further increase the complexity of epigenetic regulation by affecting their protein stability, subcellular localization and interactions with other regulatory factors [62]. For example, in yeast, the intrinsically disordered regions of HMTs are rich in phosphorylation sites, suggesting that such modifications may regulate their enzymatic activity by reshaping protein–protein interactions; in mammals, environmental stimuli such as hypoxia can link cellular metabolism with epigenetic regulation by modulating the expression level and catalytic activity of methylation-modifying enzymes [63].

The functional specificity of different histone methylation sites constitutes a complex “histone code”. H3K4me3 promotes gene expression by recruiting transcriptional activation complexes; H3K9me2/3 is involved in heterochromatin formation and transcriptional repression by binding to HP1 proteins; H3K36 methylation regulates transcriptional elongation and RNA processing; and H3K79 methylation mediated by *DOT1L* is involved in transcriptional regulation and DNA damage repair. These modifications do not exist in isolation, but integrate a variety of regulatory signals through synergistic or antagonistic effects to jointly achieve the precise regulation of dynamic changes in chromatin and gene expression [64].

The enzymatic regulatory network of histone methylation is a core component of epigenetic regulation, and its complexity stems from the functional antagonism between methyltransferases and demethylases, the multi-level regulation of modifying enzymes, and the unique biological functions carried by different methylation sites. In-depth analysis of this regulatory system not only helps clarify the physiological functions of normal cells, but also provides an important framework for elucidating the molecular mechanisms of epigenetic abnormalities in diseases such as cancer. Given the central role of abnormal histone methylation in tumor initiation and progression, intervention strategies targeting methyltransferases have become an important direction of epigenetic therapy, offering opportunities to develop novel anticancer drugs (Figure 1).

#### 2.1.2. Dysregulated Methylation Patterns Driving Malignant Phenotypes

Dysfunction of histone methyltransferases such as *KMT2C*, *KMT2D* and *DOT1L* can lead to aberrant histone methylation and has been associated with tumor initiation and progression in the models cited below. As the core catalytic subunits of the COMPASS complex, both *KMT2C* and *KMT2D* belong to the lysine methyltransferase family and mainly mediate histone H3K4 methylation, a mark that is usually enriched in the promoter regions of actively transcribed genes [65]. Gene mutations or downregulated expression of *KMT2C* and *KMT2D* can occur in a variety of solid tumors and hematological malignancies, which disrupt the normal chromatin state and gene expression, thereby interfering with key biological processes such as epithelial–mesenchymal transition (EMT), ERK/MAPK signaling, and mitochondrial function, and ultimately promoting tumorigenesis [35]. For example, *KMT2C* mutations may lead to the loss of tumor suppressor capacity and promote malignant cellular transformation. On the other hand, *DOT1L*, the only known methyltransferase that can catalyze H3K79 methylation, has been reported to be involved in tumor metabolic reprogramming; in a cadmium-induced breast cancer model, H3K79 monomethylation-mediated *PAK2* upregulation was linked to metabolic reprogramming and impaired autophagic flux [66], inducing cancer cells to form a metabolic phenotype conducive to biosynthesis and energy supply, thereby supporting their rapid proliferation and survival. Given its central role in maintaining the metabolic adaptability of tumors, *DOT1L* has become an important target for epigenetic therapy, and several selective inhibitors have entered early clinical evaluation. However, clinical activity has been modest, indicating that target engagement alone may not be sufficient and that patient selection and combination strategies remain important [67].

Aberrant histone methylation not only leads to the dysfunction of specific modifying enzymes, but also affects the proliferation, migration, and immune evasion of tumor cells. Its core mechanism lies in its ability to reshape chromatin accessibility, thereby reprogramming the transcriptional network, interfering with cell cycle regulation, and enhancing metastatic potential. For example, the significant enrichment of repressive marks such as H3K27me3 catalyzed by *EZH2*, the catalytic subunit of PRC2, and the silencing of multiple tumor suppressor genes promote an aggressive tumor phenotype [68,69]. Aberrant histone methylation can lead to immune evasion by regulating the tumor microenvironment and inhibiting the innate immune response. Deletion of the methyltransferase *NSD1* can cause a decrease in H3K36me2 levels and an increase in H3K27me3 levels, thereby inhibiting the expression of interferon-related genes, impairing the chemotaxis and infiltration ability of T cells, and ultimately promoting immune evasion [47]. This finding emphasizes that histone methylation is an important epigenetic regulatory hub connecting the complex interactions between tumor cells and the immune system.

At the molecular level, aberrant histone methylation can drive the proliferation and migration of tumor cells by reshaping the epigenetics of key signaling pathways. For example, *NRIP1*, a transcription factor highly expressed in oral squamous cell carcinoma (OSCC), targets *NSD2*, which catalyzes H3K36 methylation in the promoter region of the *DGCR8* gene and activates the transcription of downstream oncogenes, thereby accelerating tumor growth and metastasis [70]. In hepatocellular carcinoma (HCC), glutamate signaling can negatively regulate the levels of *EZH2* and H3K27me3, thereby increasing *CCL2* expression, promoting the recruitment and polarization of TAMs, and constructing an immune microenvironment conducive to tumor progression [71].

The above evidence collectively reveals that aberrant histone methylation driven by mutations or dysregulated expression of methyltransferases such as *KMT2C*, *KMT2D*, *DOT1L* and *EZH2* can activate a highly synergistic pro-tumor network. This network not only reshapes transcriptional programs and reprograms metabolic pathways, but also simultaneously enhances the proliferation and migration capacity of tumor cells and impairs antitumor immune surveillance. Against this background, small-molecule inhibitors targeting these enzymes are being investigated for their potential to reverse malignant phenotypes; clinical benefit remains to be established, and several preclinical studies and early-phase trials have provided preliminary support for these strategies. Systematic analysis of the exact molecular mechanisms of aberrant histone methylation in tumor initiation and progression may therefore inform the development of more effective epigenetic therapies, the realization of individualized intervention and the ultimate improvement of patient survival and prognosis.

#### 2.1.3. Remodeling of Histone Methylation Landscapes Under Microenvironmental Stress

The TME is composed of a variety of components including cancer cells, immune cells, cancer-associated fibroblasts (CAFs) and the extracellular matrix (ECM), which plays a key role in tumor progression and metastasis [72]. Stimuli such as hypoxia and acidosis lead to an acidic TME, which is conducive to tumor growth. In solid tumors, abnormal angiogenesis and metabolic reprogramming driven by the Warburg effect jointly lead to local hypoxia and extracellular acidification, which can directly regulate the activity of methylation-related modifying enzymes, thereby reprogramming gene expression programs and promoting the adaptation and progression of tumor cells [73,74]. For example, in breast cancer (BC), hypoxic and acidic conditions can induce the cytoplasmic translocation of the histone methyltransferase *SETDB1*, thereby enhancing glycolytic activity through the *c-MYC-LDHA* axis and driving epithelial–mesenchymal transition, endowing tumor cells with adaptive advantages for invasive metastasis and promoting disease progression [75]. In addition, in pancreatic cancer (PC), hypoxia and abnormal angiogenesis can induce the high expression of *NOX4*, thereby inhibiting the activity of the histone demethylase *KDM5A*, increasing the level of H3K4me3 modification and activating the key EMT gene *SNAIL1*, ultimately enhancing the invasive and metastatic ability of tumor cells [52]; the same conditions in ovarian cancer can activate the histone arginine methyltransferase *PRMT5*, which forms a metabolic and epigenetic regulatory loop by catalyzing the methylation modification of histones and *ENO1*, a key glycolytic enzyme, to drive the rapid proliferation of tumor cells [76].

TME-induced remodeling of histone methylation also occurs in stromal and immune compartments, although its direction and functional consequences vary with cell type and experimental model. In cancer-associated fibroblasts (CAFs), epigenetic reprogramming, including altered histone methylation, has been implicated in the regulation of activation-associated states and phenotypic heterogeneity in selected models [77]. In activated hepatic stellate cells and hepatoma cells, *ASH1L*-associated H3K4me3 deposition was reported to increase *CCL2* and *CSF1* expression, promote macrophage recruitment and induce an M2-like phenotype in a fibrosis-associated hepatocellular carcinoma model; high *ASH1L* and *CD68* expression were associated with poor prognosis in the corresponding cohort [78]. This finding links a specific methylation regulator and histone mark to macrophage recruitment in one model but does not establish a general stromal program across tumors. In renal cancer, *SETD2* inactivation was reported to reduce H3K36me3 at the *NR2F1* locus, decrease *NR2F1* transcription and increase *STAT1* expression. These changes were associated with increased CD8+ T-cell infiltration and PD-L1 expression in the tested model [79]. In a separate study, *LSD1* inhibition increased CD8+ T-cell infiltration and altered responses to immune-checkpoint blockade in selected models [80]. Together, these observations identify candidate links between histone methylation and immune-cell infiltration. However, the relevant cell population, genomic locus and metabolic state are not defined consistently, and none of these findings alone establishes a general metabolite-driven mechanism. These findings should therefore not be generalized into a single pathway through which histone methylation controls antitumor immunity.

Hypoxia, acidosis and other microenvironmental factors can alter the expression level and catalytic activity of HMTs and HDMs [63]. This can lead to dynamic rearrangement of histone methylation. In the models cited here, these changes have been reported in tumor cells, CAFs, macrophages [78] and T cells [81]. The direction and functional consequences vary by cell type, enzyme, and experimental system. These findings should therefore not be generalized into a single pathway through which microenvironmental stress controls antitumor immunity. The interaction between microenvironmental stress and histone methylation therefore constitutes the core axis of tumor adaptation and progression in the TME. Intervening in this regulatory network is being investigated as a therapeutic strategy; its clinical benefit remains to be established. However, the context dependence of histone methylation and the complexity of the regulatory network still require further in-depth research to optimize epigenetic therapy, achieve precise reprogramming of the TME; preclinical studies suggest strategies to overcome tumor drug resistance [82,83].

### 2.2. Reverse Regulatory Effect of Tumor Metabolic Reprogramming on Histone Methylation

#### 2.2.1. Metabolic Products as Methyl Donors Regulating Histone Methylation

In recent years, the bidirectional regulatory network between cellular metabolism and epigenetics has become a research hotspot. In particular, a comprehensive understanding of the interaction between metabolic reprogramming and epigenetic modifications is conducive to the development of more effective cancer therapies. The core mechanism of their cross-regulation lies in the ability of intracellular metabolites to act as methyl donors to directly participate in and regulate histone methylation, thereby affecting the spatial conformation of chromatin and the transcriptional expression of downstream genes [84]. Among them, S-adenosylmethionine (SAM), a universal methyl donor in organisms, is an indispensable cofactor for maintaining the catalytic activity of DNA and histone methyltransferases [85]. SAM synthesis mainly depends on the methionine cycle, which is closely coupled with one-carbon metabolic pathways such as the folate cycle. These pathways can integrate one-carbon units produced by the metabolism of amino acids such as serine and glycine to provide sufficient raw materials for methionine regeneration and subsequent SAM synthesis [86]. In cancer cells, the dynamic fluctuation of SAM levels is closely related to the changes in the demand for methylation reactions, and these methylation modifications are directly involved in multiple key processes of tumor biology such as oncogene activation, tumor suppressor gene silencing, and metabolic enzyme activity regulation.

The high dependence of the methionine cycle on one-carbon metabolism further highlights the core regulatory position of the serine-glycine-one-carbon (SGOC) metabolic axis in maintaining the dynamic balance of histone methylation modifications. Serine and glycine provide one-carbon units through tetrahydrofolate intermediates, thereby supporting methionine regeneration and SAM synthesis [87]. Blockade of this pathway, whether due to the lack of nutrient substrate supply or the dysfunction of key enzymes, will lead to the disorder of histone methylation patterns, thereby potentially facilitating tumor initiation and progression. For example, in the *SLC43A2* model, tumor cells reduce methionine and SAM levels in CD8+ T cells, leading to reduced H3K79me2 at the *STAT5* locus and impaired T-cell effector function [88]. Folate-dependent one-carbon metabolism regulates epigenetic modifications such as DNA and histone methylation in cancer cells by providing methyl donors, and the abnormal expression of key enzymes such as *MTHFR* and *SHMT2* and the folate receptor (*FOLR1*) can cause epigenetic disorders, thereby promoting tumorigenesis [89].

In addition to SAM, α-KG is also a key cofactor regulating the activity of histone demethylases, which mainly targets HDMs containing the Jumonji C (JmjC) domain [90]. α-KG-dependent dioxygenases can catalyze the removal of histone methyl groups, constructing a feedback pathway between cellular metabolism and epigenetic remodeling. In tumor cells, the level of α-KG fluctuates with changes in the activity of the tricarboxylic acid (TCA) cycle, and this fluctuation can directly regulate the catalytic function of histone demethylases, thereby affecting the gene expression network related to tumor cell metabolic reprogramming and abnormal proliferation [91]. Such metabolic-epigenetic feedback loops are not unique to tumor cells but also exist in *Plasmodium*. This cross-species example illustrates a conserved principle, but it does not provide direct evidence for a tumor-specific mechanism. The competitive binding of histone methyltransferases and phospholipid biosynthetic enzymes to SAM can synergistically regulate the state of histone methylation modifications and the expression pattern of sex differentiation-related genes. This cross-species conserved phenomenon further highlights the extensive biological value of methyl donor metabolism in epigenetic regulation.

The dynamic balance between SAM and its metabolic byproduct S-adenosylhomocysteine (SAH) is a key factor determining the cellular methyltransferase capacity. SAH inhibits various methyltransferases; the intracellular SAM/SAH ratio has therefore been widely used as an indicator of cellular methylation capacity [92,93]. In non-tumor systems, the SAM/SAH ratio has been reported to reflect cellular methylation capacity better than individual SAM or SAH levels, and its association with subclinical atherosclerosis was stronger in populations at high cardiovascular risk [94]. This evidence is indirect and hypothesis-generating for the tumor context; it supports the biochemical principle that the SAM/SAH ratio reflects methylation capacity, but it does not establish a role for SAM/SAH in tumor histone methylation.

The interaction between metabolism and epigenetic regulation may have clinical implications. For example, dietary nutrients involved in one-carbon unit metabolism, such as folic acid, vitamin B12, vitamin B6, methionine, choline and betaine can affect epigenetic modifications by regulating the biosynthesis of SAM, and potentially influence disease susceptibility and disease progression. Based on this, nutritional intervention to target and regulate the above metabolic pathways is being investigated as a potential strategy for the dynamic balance of epigenetics in cancer and other diseases [93,95]. In addition, targeted intervention strategies for enzymes related to methyl donor metabolism or histone methylation regulatory factors are being investigated for their potential clinical applications; for example, in a single-arm Phase II study in relapsed/refractory adult T-cell leukemia-lymphoma, valemetostat produced an ORR of 48% (5 CR, 7 PR). This study was not designed to establish survival benefit, and the results should be interpreted as activity in a defined cohort rather than as an established improvement in prognosis [96].

SAM availability can influence methyltransferase activity, whereas α-KG availability can affect α-KG-dependent demethylases. The direction and magnitude of these effects vary with the enzyme, genomic locus, and cellular state; they should not be generalized across tumor types. Where the evidence is associative rather than mechanistic, this distinction should be stated explicitly. These context-dependent relationships motivate further investigation of the histone methylation–metabolism axis (Table 2).

#### 2.2.2. KMT2D-Deficient Melanoma: Enhancer Remodeling, Glycolysis and IGF Signaling

In addition to metabolites influencing histone methylation, histone methylation can itself regulate metabolic gene expression, although its effects depend on the specific mark, genomic location, and cellular state. A defined example is *KMT2D*-deficient melanoma. In the reported melanoma models, *KMT2D* loss was associated with remodeling of H3K4me1-marked enhancers and reduced IGFBP5 expression. Reduced *IGFBP5* was linked to increased *IGF1R–AKT* signaling and higher expression of glycolysis-related genes. The study reported that glycolysis inhibition reduced the proliferation of *KMT2D*-deficient cells, and it identified *IGF1R* inhibition as a candidate vulnerability [34]. This example links enhancer-associated H3K4me1 to glycolytic reprogramming in a defined melanoma setting. It does not establish a general role for H3K4 methylation in activating glycolysis across cancers, nor does it establish IGF1R inhibition as a broadly applicable therapeutic strategy without validation in independent models. Such context dependence is consistent with the broader view that epigenetic states can shape metabolic gene expression and drug responses, although the direction varies by tumor type and treatment [101].

#### 2.2.3. Positive Feedback Loop Between H3K27me3 Modification Mediated by SUZ12 and Lactate Metabolism in Tumor Remodeling

*SUZ12*, as a core subunit of the polycomb repressive complex 2 (PRC2), is highly expressed in retinoblastoma and contributes to tumor remodeling through H3K27me3-mediated repression thereby forming a positive feedback loop with lactate metabolism. Mechanistically, excessive histone lactylation (e.g., H3K18la) in tumor cells can target the promoter region of *SUZ12* to promote its transcription, and *SUZ12* recruits the PRC2 complex to the promoter region of the tumor suppressor gene *KLF4*, silencing its expression through H3K27me3 modification. The downregulation of *KLF4* relieves the transcriptional inhibition of lactate dehydrogenase A (*LDHA*), accelerates the glycolysis process and lactate accumulation, and the excessive lactate further enhances the level of histone lactylation. This feedback loop strengthens the metabolic reprogramming of tumor cells and promotes tumor progression. Functional verification shows that targeted silencing of *SUZ12* can significantly inhibit tumor growth, and this regulatory mechanism provides an important theoretical basis for developing combined epigenetic–metabolic therapies [102] (Figure 2).

#### 2.2.4. Crosstalk Between Histone Methylation and Adjacent Epigenetic Mechanisms

This section discusses histone lactylation, non-histone methylation, RNA-associated methylation, and autophagy only where a defined molecular or metabolic link to histone methylation can be identified. Each mechanism is included because it regulates a node in the histone methylation–metabolism axis, not because it shares a general metabolic context with histone methylation. Other aspects of these mechanisms are not discussed. Histone lactylation is a lactate-derived post-translational modification that is chemically and mechanistically distinct from histone methylation. It is discussed here because lactate-derived lactylation can directly regulate the expression or activity of histone methylation regulators. In retinoblastoma, H3K18la at the *SUZ12* promoter increases *SUZ12* expression, enabling PRC2-mediated H3K27me3 deposition at the *KLF4* locus; *KLF4* repression then increases *LDHA* expression and lactate production, forming a positive feedback loop between lactylation and histone methylation. This circuit is model-specific and should not be generalized to other tumors. Outside this defined intersection, histone lactylation is not a form of histone methylation and is not discussed further.

Non-histone methylation is included only where a methyltransferase that also modifies histones directly regulates a metabolic enzyme or a metabolite pool relevant to histone methylation. *CARM1* can methylate both histone and non-histone substrates and has been implicated in lipid metabolism, autophagy, and redox homeostasis in cancer-related models [103,104]. However, the evidence does not establish a direct effect on SAM, α-KG, or histone methylation; it is included only because *CARM1* can regulate metabolic enzymes or metabolite pools relevant to histone methylation. *SUV39H1*-mediated methylation of *FASN* has been associated with *FASN* ubiquitination and degradation in a dental pulp stem-cell model [105]. This finding is included only as evidence that non-histone methylation can regulate a metabolic enzyme; it is not direct evidence for a general tumor-metabolic mechanism. It should therefore be regarded as evidence that non-histone methylation can regulate metabolic enzymes, but not as direct evidence that *SUV39H1-FASN* signaling is a general tumor-metabolic mechanism. This finding is included only as evidence that non-histone methylation can regulate a metabolic enzyme; it does not establish *SUV39H1-FASN* signaling as a general tumor-metabolic mechanism. In addition, *PRMT5* can methylate non-histone substrates involved in RNA splicing. In prostate and pancreatic cancer models, *PRMT5* methylates Sm protein family members to promote snRNP assembly, which supports correct splicing of mRNAs encoding metabolic enzymes such as *FASN* and *GLUT1* [60,106]. This pathway may indirectly affect the expression of metabolic enzymes and, in principle, alter the availability of metabolites that regulate histone methyltransferases and demethylases. However, the cited studies did not measure SAM or α-KG levels, nor did they demonstrate changes in histone methylation in these models. This evidence is therefore indirect and hypothesis-generating, and the direction of any feedback is likely to depend on the specific PRMT, substrate, tumor type, and cellular state. This section is included only because these enzymes can regulate metabolic enzymes or metabolite pools that feed into the histone methylation machinery; other non-histone methylation events are not discussed. It is discussed here only because *PRMT5* can regulate metabolic enzymes whose products feed into the histone methylation machinery.

Methylation-dependent regulation of autophagy is not discussed as a core mechanism in this review. Autophagy can influence nutrient recycling and mitochondrial metabolism, and may therefore affect intracellular pools of SAM, α-KG and other metabolites that serve as substrates or cofactors for histone-modifying enzymes. *CARM1*-dependent pathways have been associated with changes in autophagic flux in cancer-related models [103,107]. However, the cited studies did not measure SAM or α-KG levels, nor did they demonstrate that autophagy directly alters histone methylation in tumor cells. The relationship between autophagy and histone methylation should therefore be considered indirect and context-dependent, rather than a universal mechanism by which autophagy controls histone methylation.

Collectively, histone lactylation, non-histone methylation, RNA-associated methylation and autophagy represent adjacent regulatory layers that can intersect with histone methylation through shared enzymes, overlapping metabolic inputs or convergent transcriptional programs. Current evidence supports mechanistic crosstalk in selected experimental systems, but does not establish a universal or necessarily synergistic relationship across tumors. Future studies should define the relevant substrates, cellular compartments, metabolic conditions, and causal feedback loops before these pathways can be evaluated as therapeutic targets.

These mechanisms are inherently context-dependent and are often inferred from bulk tumor samples. Because metabolites such as SAM, α-KG, lactate, and 2-HG are shared across tumor cells, immune cells and stromal cells, bulk transcriptomic or epigenomic data cannot easily distinguish cell-intrinsic regulation from cell-composition effects. In addition, the same histone methylation mark can have different functional consequences depending on cell type, locus, and metabolic microenvironment. Resolving these complexities requires computational deconvolution, single-cell epigenomics and spatially resolved profiling, which are discussed in Section 4.4.

## 3. The Role of Histone Methylation in Tumor Immunometabolism

### 3.1. Regulation of Tumor Immune Cell Metabolic Reprogramming by Histone Methylation

Histone methylation provides an important link between nutrient availability, metabolic state, and immune-cell function in the tumor microenvironment (TME). By regulating genes involved in glycolysis, oxidative phosphorylation (OXPHOS), fatty-acid oxidation (FAO), cytokine production and antigen presentation, histone methyltransferases and demethylases can influence the functional state of tumor-infiltrating immune cells. However, these effects are strongly dependent on cell type, differentiation state, inflammatory stimulation, and local nutrient conditions.

TAMs provide a representative example of this context dependence. In a hepatocellular carcinoma (HCC) model, *EZH2* loss reduced H3K27me3 and was reported to promote a polarization state that the authors described as M2-like. This state was not resolved at the metabolic or marker level, and the finding should not be generalized to a universal TAM program. More broadly, TAMs are heterogeneous and plastic; their states are shaped by hypoxia, lactate, cytokines, lipids, and tissue location and do not map consistently onto a binary M1/M2 framework [108]. Links between a specific histone mark and glycolysis, oxidative phosphorylation or fatty-acid oxidation should therefore be described in relation to the macrophage state and model in which they were demonstrated. The effect of a particular histone methylation event should therefore be interpreted together with the macrophage state, tissue location and metabolic environment in which it occurs.

Similar coupling between histone methylation and immune function has been reported in dendritic cells (DCs). Stimulus-dependent changes in H3K4 methylation have been associated with the expression of genes involved in antigen processing, antigen presentation, and cellular metabolism in experimental models. The direction and magnitude of these effects vary according to the activating stimulus and the developmental state of the DCs. Whether *KMT2D* specifically contributes to these effects in DCs remains to be established; current evidence supports a more general role for H3K4 methylation in this context [109].

CD8+ T cells illustrate how tumor-cell nutrient competition can directly affect histone methylation in immune cells. Tumor cells expressing the methionine transporter *SLC43A2* can reduce extracellular methionine availability and thereby limit methionine uptake by neighboring CD8+ T cells. This decreases intracellular S-adenosylmethionine (SAM), the methyl donor required for several histone methyltransferases. Reduced SAM availability impairs *DOT1L*-dependent H3K79me2/3 deposition at the *STAT5* locus, resulting in lower *STAT5* expression and compromised T-cell effector function [88,110]. This mechanism provides a direct link between tumor-cell methionine consumption, histone methylation and CD8+ T-cell dysfunction.

The biological consequences of *DOT1L* inhibition, however, are context dependent. Although H3K79me2 under methionine-restricted conditions is associated with impaired effector activity, studies in mature CD8+ T cells have reported that loss of *DOT1L* activity can promote memory-like or innate-like features and increase intrinsic cytotoxicity under particular experimental conditions [110]. These observations do not necessarily contradict the methionine–*DOT1L–STAT5* mechanism. Instead, they indicate that *DOT1L* activity may have different effects depending on T-cell differentiation, activation state, antigen exposure, and metabolic context. Accordingly, changes in H3K4me3 or H3K9me3 should not be attributed to methionine competition unless they have been directly demonstrated in the relevant experimental system [88].

Together, these findings indicate that histone methylation connects immune-cell metabolism with immune function in a cell-type- and context-dependent manner. The same metabolic input may produce different epigenetic and functional outcomes in TAMs, DCs, and T cells, which should be considered when interpreting tumor immunometabolic studies.

### 3.2. Metabolic-Epigenetic Axis in Tumor Immune Evasion

Tumor metabolic reprogramming contributes to immune evasion by altering the availability of methyl donors, cofactors, and inhibitory metabolites. These metabolic changes can affect histone methyltransferases and demethylases directly or indirectly, thereby modifying chromatin accessibility, gene expression, and immune-cell activity. Four representative metabolic inputs—lactate, D-2HG, arginine and methionine—are discussed below.

#### 3.2.1. Metabolite-Mediated Regulation of Histone Methylation and Immune Suppression

Lactate is not only a glycolytic end product but also a signaling metabolite that can influence chromatin regulation. Lactate can induce histone lactylation, a post-translational modification that is chemically and mechanistically distinct from histone methylation. Histone lactylation may activate genes involved in the differentiation and function of immunosuppressive cell populations, including TAMs and regulatory T cells. In parallel, the lactate–*SUZ12*–H3K27me3 positive-feedback loop described in Section 2.2.4 illustrates how lactate signaling may interact with the histone methylation machinery. These interconnected pathways have been associated with T-cell exhaustion and increased immune-checkpoint expression in experimental models [111,112,113,114]. Histone lactylation should therefore be discussed as a related but distinct regulatory layer rather than as a form of histone methylation.

In IDH-mutant tumors, the oncometabolite D-2-hydroxyglutarate (D-2HG) competitively inhibits α-KG-dependent JmjC histone demethylases. This inhibition can cause aberrant accumulation of repressive histone methylation marks and suppress the transcription of genes involved in differentiation, antigen presentation, and immune recognition. The resulting chromatin state may contribute to an immune-excluded or immunosuppressive TME. However, the magnitude of this effect depends on D-2HG concentration, tumor genotype, cellular compartment, and the relative expression of individual demethylases [9,11].

Arginine depletion represents another metabolic mechanism of immune suppression. Tumor cells and myeloid-derived suppressor cells can consume or degrade extracellular arginine, reducing arginine availability for infiltrating T cells. Arginine limitation has been reported to reduce T-cell proliferation, impair cytokine production and promote chromatin compaction or reduced chromatin accessibility. These effects may restrict transcription-factor binding and compromise the activation of antitumor T-cell programs [115]. Because the relationship between arginine availability and specific histone methylation marks remains model dependent, the mechanism should be described as a metabolic influence on chromatin accessibility and histone-regulatory pathways rather than as a universal methylation mechanism.

Altered methionine metabolism can affect histone methylation by changing the intracellular SAM pool. Tumor-cell competition for methionine can consequently impair histone methylation in neighboring immune cells and promote T-cell dysfunction. The methionine–*SLC43A2*–SAM–*DOT1L*–H3K79me2–*STAT5* pathway described in Section 3.1 is a representative example of this process [88,116]. To avoid repetition, the detailed molecular mechanism is presented only in Section 3.1, whereas this section emphasizes methionine availability as a broader metabolic determinant of epigenetic regulation.

Collectively, these examples show that metabolic stress can regulate immune function through direct effects on chromatin-modifying enzymes, altered cofactor availability, and indirect remodeling of histone-regulatory networks. The resulting immune phenotype is determined by the interaction between the metabolite, the target cell, and the local tissue context (Figure 3).

#### 3.2.2. Histone Methylation in Immune-Checkpoint Regulation and Immunotherapy Response

Histone methylation at promoters and enhancers of immune-checkpoint and immune-effector genes may influence PD-L1 expression, antigen presentation and T-cell exhaustion. At the *CD274* locus, activating marks such as H3K4me3 and H3K27ac may support transcription in some tumor contexts, whereas repressive marks such as H3K27me3 and H3K9me3 may restrict expression. These associations are not universal, because the effect of a particular mark depends on transcription-factor occupancy, chromatin accessibility, tumor lineage and inflammatory signaling.

Dysregulation of histone-modifying enzymes in tumor cells can therefore affect immune-checkpoint expression and antigen presentation. In addition, histone methylation in immune cells may alter the expression of cytokines, exhaustion-associated genes and cytotoxic-effector molecules. Metabolic inputs such as lactate, methionine limitation and D-2HG accumulation can reshape these chromatin states by changing lactylation, methyl-donor availability, or demethylase activity [117].

These mechanisms provide a biological rationale for combining epigenetic or metabolic interventions with immune-checkpoint blockade [118]. Nevertheless, molecular association, cellular activity and therapeutic efficacy should be clearly distinguished. Current evidence ranges from mechanistic studies and cell-culture experiments to animal models and early-phase clinical trials; improved checkpoint expression or immune-cell activation in a preclinical model should not be presented as proof of clinical benefit.

The metabolic–epigenetic interactions described in this section are likely to vary across spatial niches within the tumor microenvironment. Lactate-rich, methionine-depleted, or 2-HG-accumulating regions may coexist with nutrient-replete areas, and immune cells in different niches may experience distinct epigenetic pressures. Bulk tumor data cannot capture this spatial heterogeneity, and even single-cell dissociation-based methods lose tissue architecture and cell–cell neighborhood information. Spatial transcriptomics, spatial metabolomics, and multiplex imaging are therefore needed to determine whether metabolically stressed niches co-localize with specific histone methylation marks and dysfunctional immune-cell states. These approaches, together with the computational tools required to integrate them, are discussed in Section 4.4.

### 3.3. Potential of Combined Application of Histone Methylation-Targeted Therapy and Tumor Immunotherapy

Histone methylation-targeted agents are being investigated in combination with immunotherapy because histone methylation can regulate tumor-cell metabolism, antigen presentation, inflammatory signaling, and immune-cell function. The therapeutic effects of these combinations are likely to depend on tumor genotype, immune contexture, treatment sequence, and the specific epigenetic dependency of each tumor. Most of the mechanisms discussed below have been established primarily in cell-line and animal models, and the clinical benefit of combining these agents with immune-checkpoint blockade has not yet been established.

*EZH2* inhibition can relieve H3K27me3-mediated repression of selected immune-related genes and has been reported to increase immune-cell infiltration or improve sensitivity to PD-1/PD-L1 blockade in selected preclinical models [119]. However, *EZH2* inhibition can also produce context-dependent effects on T cells and regulatory T cells, and its combination with immunotherapy should therefore be evaluated in relation to both tumor and immune compartments. *EHMT1/GLP* and *EHMT2/G9a* catalyze H3K9 methylation; inhibition of these enzymes can reduce H3K9me2, activate selected endogenous-retroviral or DNA-sensing pathways, and increase the response to anti-PD-1 therapy in selected tumor models [120]. *PRMT5* inhibition may influence immune-related transcription, RNA splicing and cellular metabolic programs, providing a rationale for combination with immune-checkpoint blockade in selected models [121], although *PRMT5* has important functions in both malignant and normal cells, and systemic inhibition may cause toxicity or produce opposing effects in different immune-cell populations.

*LSD1/KDM1A* inhibition can increase MHC-I antigen presentation and enhance the expression of endogenous tumor antigens in selected tumors, potentially sensitizing tumors to immune-checkpoint blockade when baseline antigen presentation is defective [122]. The response is not expected to be uniform across all tumor types, and the relevant histone and non-histone substrates should be defined experimentally. *LDHA*, by contrast, is a metabolic rather than a histone-modifying enzyme; inhibition of *LDHA* or lactate production may reduce lactate-driven immune suppression and complement epigenetic therapy in selected contexts [123], but this strategy should be presented as a metabolic co-targeting approach rather than as direct inhibition of histone methylation.

Looking forward, several strategies may help translate these mechanistic insights into clinical practice. CRISPR/dCas9-mediated epigenetic editing has been used for locus-specific regulation of histone marks in proof-of-concept studies, including non-tumor systems [124]; translation to tumor immunity remains to be established. Biomarker-guided patient stratification based on epigenetic and metabolic characteristics may help identify patients most likely to benefit from combination regimens and reduce unnecessary toxicity. In addition, combining epigenetic drugs with immune checkpoint inhibitors, adoptive cell therapy and cancer vaccines may help address tumor heterogeneity and immune evasion, but these combinations should be evaluated in appropriately designed clinical trials rather than assumed to be broadly synergistic.

Overall, the combination of histone methylation-targeted therapy with immunometabolic modulation remains a promising but unproven strategy. Future studies should define predictive biomarkers, treatment sequence, dose-limiting toxicities, and the effects of these agents on different immune-cell populations before their clinical utility can be determined (Table 3) (Figure 4).

## 4. Research Progress of Therapeutic Strategies Related to Histone Methylation and Tumor Metabolism

### 4.1. Research and Development and Application of Histone Methyltransferase Inhibitors

Given the central role of histone methyltransferases (HMTs) in epigenetic regulation and tumor initiation and progression, the research and development of small-molecule inhibitors targeting HMTs such as *DOT1L* and *KMT2C/D* has become a research hotspot in the field of tumor therapy. *DOT1L* can specifically catalyze the methylation modification of histone H3 lysine 79 (H3K79), and this modification process is closely related to oncogene transcription and diseases such as *MLL*-rearranged leukemia. Advances in structure-guided drug design have driven the synthesis of non-nucleoside compounds that can mimic the structural characteristics of the S-adenosyl-L-methionine (SAM) cofactor to achieve selective inhibition of *DOT1L* activity. For example, derivatives containing an aminopyrimidine group and combined with a functionalized aryl group have been confirmed by in vitro structure–activity relationship studies to not only have potent *DOT1L* inhibitory activity but also show good selectivity for other methyltransferases [127]. Studies have shown that novel compounds with a quinazoline skeleton substituted with 2-tetrahydroisoquinoline can not only enhance the enzyme inhibitory effect on *G9a* but also exhibit significant anti-proliferative activity in a variety of cancer cell lines, such as colon cancer. Mechanistically, such inhibitors can reduce the level of H3K9me2 and trigger autophagy and apoptosis of cancer cells by inducing the production of reactive oxygen species (ROS), showing good antitumor efficacy and safety in vivo [128]. In addition, protein arginine methyltransferases (PRMTs), especially *PRMT5* and *PRMT1*, have also become popular therapeutic targets in the HMT family due to their key roles in transcriptional regulation, RNA splicing, and oncogenic signal transduction. The research and development of *PRMT5* inhibitors covers a variety of mechanisms of action, including SAM-competitive inhibitors, substrate-competitive modulators, and PROTAC-based degraders. These inhibitors have different tumor specificity and toxicity characteristics, and some candidate drugs have entered clinical trials, showing good clinical translational potential [129,130]. At present, the research and development of inhibitors targeting other methyltransferases such as *NSD3* and *SETD8* is also progressing actively, but many challenges still remain in terms of drug specificity and selectivity [40,131]. In addition, to overcome the limitations such as drug resistance and side effects of single-target drugs, the research and development of dual-target inhibitors that simultaneously inhibit *EZH2* and other epigenetic regulators has entered the exploration stage [132]. These advances in medicinal chemistry and drug discovery provide a basis for developing histone methyltransferase-targeted epigenetic therapies (Table 4).

Regarding the therapeutic effect of histone methyltransferase inhibitors in different tumor types, clinical and preclinical studies have demonstrated the heterogeneity of treatment responses, which are jointly determined by the tumor-specific epigenetic landscape and microenvironmental factors. For example, *EZH2* inhibitors have shown efficacy in hematological malignancies but have limited efficacy when applied to solid tumors, partly because the hypoxic tumor microenvironment can regulate *EZH2* function through interaction with hypoxia-inducible factors, thereby reducing drug efficacy [63]. In non-small cell lung cancer (NSCLC), the EHMT2 inhibitor BIX01294 can induce autophagy-mediated cell death by regulating the cholesterol biosynthesis pathway, which suggests that different tumor types have exploitable metabolic vulnerabilities [148]. Similarly, in pancreatic ductal adenocarcinoma, BIX01294 inhibits tumor growth by disrupting the glutaminase-mediated glutathione dynamic balance, further highlighting the interaction of the metabolic-epigenetic regulatory axis in cancer therapy [141]. The drug resistance mechanism of histone methyltransferase inhibitors is complex and usually related to epigenetic plasticity and the activation of compensatory pathways. For example, random demethylation and redundant epigenetic inhibition mechanisms lead to the heterogeneity of cancer cell responses to DNA methyltransferase inhibitors, which indicates that the combined application of histone deacetylase inhibitors and methyltransferase inhibitors may help improve clinical efficacy [149]. In addition, acquired resistance may also result from metabolic reprogramming and its cross-talk with epigenetic modifications, which requires the research and development of next-generation inhibitors and the optimization of combination therapy regimens [45,150,151]. Preclinical studies have investigated whether combining epigenetic drugs with immunotherapy or targeted therapy can overcome resistance, but clinical benefit remains to be established [152]. Despite the above progress, problems such as limited selectivity, off-target effects and toxicity remain major obstacles. At present, a number of studies are committed to solving these limitations and optimizing the therapeutic window by means of structure-guided design, covalent inhibitor strategies and artificial intelligence-driven drug repurposing technology [153,154]. In summary, histone methyltransferase inhibitors are a rapidly developing class of epigenetic therapeutic drugs that have shown antitumor activity in a variety of malignant tumors, but the key to their clinical success lies in overcoming tumor heterogeneity and drug resistance mechanisms through rational drug design and combination therapy strategies.

### 4.2. Epigenetic Remodeling via Metabolic Intervention Strategies

Metabolic regulatory drugs can significantly affect the state of histone methylation, and this regulatory effect is mainly achieved by regulating key metabolites that are cofactors or substrates of histone methyltransferases and demethylases. α-KG is a representative example, which is not only an intermediate product of the tricarboxylic acid (TCA) cycle but also an essential cofactor for histone demethylases containing the Jumonji C (JmjC) domain. Changes in α-KG availability will directly affect histone demethylase activity, thereby affecting the gene expression program related to tumor metabolism and progression. For example, in glioma, isocitrate dehydrogenase (IDH) mutations lead to the massive accumulation of the oncometabolite D-2-hydroxyglutarate (D-2HG), which can competitively inhibit α-KG-dependent demethylases, resulting in extensive histone hypermethylation and an aberrant epigenetic landscape, thereby promoting tumorigenesis. Therapeutic strategies aimed at restoring α-KG levels or inhibiting mutant IDH enzymes (e.g., vorasidenib) have shown therapeutic potential in reversing the above epigenetic abnormalities and inhibiting tumor growth [155]. Another key metabolite is S-adenosylmethionine (SAM), a universal methyl donor for histone methyltransferases. Regulating SAM synthesis or availability by targeting methionine metabolism or methionine adenosyltransferase 2A (*MAT2A*) may alter histone methylation patterns. In a lung cancer model, *MAT2A* inhibition downregulated specific histone methylation marks such as H3K9me2 and H3K36me3, and methionine deficiency more broadly reduced H3K4me3, H3K9me2, H3K27me3 and H3K36me3, thereby reversing the chemoresistance of cisplatin-resistant cells [156]. In addition, a multiplex methionine-modulating hydrogel combined with a methionine metabolism inhibitor can effectively reduce SAM production and histone methylation levels in a variety of cancer models, induce immunogenic cell death, and enhance antitumor immune responses [157]. In addition to α-KG and SAM, other metabolic intermediates such as β-hydroxybutyrate (BHB) have also been shown to have epigenetic modification functions, which can act as substrates for histone β-hydroxybutyrylation and affect histone methylation, linking metabolic states such as ketosis with epigenetic regulation in cancer and other diseases [158]. In addition, metformin, an anti-diabetic drug widely used in clinical practice, has been confirmed to inhibit the histone methyltransferase *CARM1*, downregulate the level of histone H3 arginine methylation and inhibit the expression of gluconeogenic genes, which further highlights the mechanism by which metabolic drugs directly regulate epigenetics [159]. The above research results collectively confirm that metabolite availability and metabolic enzyme activity play a core role in shaping the histone methylation landscape. Drug regulation of these metabolic-epigenetic axes through metabolic intervention provides an attractive new approach for cancer therapy, which is expected to achieve the reprogramming of the abnormal epigenetic state of tumors.

Studies on histone methyltransferase inhibitors (e.g., *G9a* inhibitors) have further confirmed the therapeutic potential of combined targeting of metabolism and epigenetics. In hepatoblastoma, *G9a* is highly expressed and can synergistically regulate oncogenic metabolic reprogramming. Studies have demonstrated that pharmacological inhibition of *G9a* can not only inhibit tumor growth but also disrupt the metabolic pathways essential for tumor cell survival. This finding illustrates how metabolic regulation and histone methylation can intersect in cancer [160]. Similarly, protein arginine methyltransferase 5 (*PRMT5*) is closely associated with the initiation and progression of a variety of malignant tumors, and small-molecule inhibitors targeting this enzyme have now entered clinical trials, which also directly reflects the clinical translational potential of epigenetic enzyme-targeted regulation [129]. These epigenetic drugs may act synergistically with metabolic modulators to further improve treatment effects. Importantly, the dynamic interaction between metabolism and histone methylation has significant context dependence, and its regulatory mode may vary with tumor types and microenvironmental conditions (e.g., hypoxia, acidosis), which in turn can affect histone modifications and metabolic enzyme activity. In-depth analysis of the metabolic regulatory mechanism of histone methylation can therefore provide a core mechanistic basis for developing novel combined therapeutic strategies that utilize tumor metabolic vulnerabilities and simultaneously correct epigenetic disorders.

Clinically, a number of trials are actively exploring epigenetic drugs targeting histone methylation enzymes, and such drugs are usually used in combination with metabolic therapy or standard chemotherapy. For example, the clinical evaluation of *PRMT5* inhibitors focuses on their ability to overcome tumor drug resistance and regulate tumor metabolism [161]. Combining metabolite supplementation or deprivation strategies with epigenetic drugs is being investigated in preclinical models; whether this improves treatment response in patients remains to be determined (Table 5).

### 4.3. Combination Therapy Strategies and Future Outlook

The studies reviewed above indicate that combining histone methylation modulators with metabolic interventions, immunotherapy, chemotherapy or radiotherapy can target complementary tumor vulnerabilities. Collectively, these preclinical and early translational findings support rational combination design, but their benefit remains context-dependent and has not been established as a universal therapeutic effect [165].

Future research should translate these mechanistic insights into biomarker-guided combination regimens. Priority should be given to defining the tumor and microenvironmental states that predict sensitivity, establishing pharmacodynamic biomarkers, and determining how treatment sequence and dose influence response. Artificial intelligence and machine-learning models may help prioritize drug combinations, dosing schedules, and biomarker-defined patient subgroups from integrated molecular data [166], but their predictions must be tested in functional experiments and prospective clinical studies. The multi-omics and spatial approaches used to characterize these states are discussed in Section 4.4.

Translation will require careful management of off-target effects and toxicity, adaptive pathway rewiring and acquired resistance, as well as substantial interpatient and intratumoral heterogeneity. The interactions among metabolism, histone methylation and immunity are bidirectional and context-dependent. It is therefore difficult to infer causality from bulk correlations. Metabolites such as S-adenosylmethionine, α-ketoglutarate, acetyl-CoA, and lactate can influence multiple enzymes and cell populations simultaneously [167]. Biomarker-driven trials with pharmacodynamic readouts, serial tissue or liquid-biopsy sampling and rational dose or sequence optimization will therefore be essential for distinguishing durable combination benefit from transient responses and for defining which patients are most likely to benefit.

### 4.4. Systems Biology and Spatial Omics Perspectives on the Histone Methylation–Metabolism–Immunity Axis

This section focuses on the analytical framework needed to resolve the mechanisms underlying the therapeutic priorities outlined in Section 4.3. A practical workflow for evaluating metabolic-epigenetic interactions computationally would be as follows: (i) deconvolve bulk RNA-seq and epigenomic data to estimate cell-type-specific signals; (ii) integrate scRNA-seq with scATAC-seq or scCUT&Tag to link metabolic states with histone marks; (iii) use spatial transcriptomics and spatial metabolomics to localize metabolite-rich or nutrient-depleted niches; (iv) infer ligand–receptor communication and regulatory networks; and (v) validate computationally predicted interactions with isotope tracing, locus-specific CUT&Tag or ChIP-qPCR, CRISPR-mediated perturbation, and functional assays in relevant immune or tumor cell models. Bulk and single-cell sequencing provide complementary information but have important limitations for resolving metabolic-epigenetic crosstalk in the tumor microenvironment. Bulk RNA-sequencing, chromatin-accessibility, and epigenomic datasets combine signals from tumor, immune and stromal cells. As a result, differences in cell-type composition can be mistaken for cell-intrinsic regulation. Reference-based deconvolution methods, such as CIBERSORTx, MuSiC and BayesPrism, can be used to estimate cell-type-specific signals when appropriate single-cell reference datasets are available [168]. Metabolic pathway scores for glycolysis, serine-glycine-one-carbon metabolism, the methionine cycle, the tricarboxylic acid cycle and fatty-acid oxidation can then be integrated with the expression of histone methyltransferases, demethylases and metabolic enzymes. These analyses should be repeated using alternative reference datasets and deconvolution methods to assess their robustness. scRNA-seq can further define transcriptional and metabolic states at single-cell resolution, but tissue dissociation generally removes spatial information and cell–cell neighborhood relationships.

Single-cell epigenomic technologies can provide a more direct link between metabolic states and chromatin regulation. scATAC-seq measures cell-type-specific chromatin accessibility, whereas scCUT&Tag or, where technically feasible, scChIP-seq can profile histone marks such as H3K4me3, H3K27me3 and H3K9me3 [169,170]. These datasets can be integrated with scRNA-seq using frameworks such as ArchR [171], Signac or latent-factor models such as MOFA+ [172]. Peak-to-gene linkage and regulatory-network analysis can then connect histone marks or accessible regulatory elements with genes involved in glycolysis, methionine metabolism, immune checkpoints and T-cell exhaustion. Prior-knowledge networks can additionally connect metabolites such as SAM, α-KG, succinate, fumarate and lactate with the enzymes and histone modifications that they regulate. Bayesian models, regularized graphical models or network-inference approaches can be used to identify context-specific regulatory relationships. These computational predictions should be considered hypotheses rather than proof of causality. Spatial transcriptomics and spatial metabolomics can add the anatomical dimension by identifying metabolite-rich or nutrient-depleted niches and determining whether they co-localize with altered expression of histone-modifying enzymes, histone marks and dysfunctional immune-cell states. Spatial-domain and neighborhood tools such as Squidpy [173], Giotto [174] and SpaGCN [175] can define tumor, immune-exclusion and tumor-boundary niches, while CellChat [176] or NicheNet can be used to infer ligand–receptor-mediated communication between metabolically stressed tumor cells, macrophages, dendritic cells and T cells.

Several limitations must be considered when interpreting these analyses. Spatial transcriptomics and mass-spectrometry imaging differ in resolution, sensitivity and molecular coverage, which complicates cross-modal registration and quantitative comparison [177]. Most spatial assays also provide a single-time-point snapshot and cannot determine the direction of a regulatory relationship. Sparse single-cell chromatin measurements, batch effects and changes induced by tissue dissociation may further affect the results [178]. Computationally inferred metabolic–epigenetic interactions should therefore be validated using isotope tracing [179]; locus-specific CUT&Tag or ChIP-qPCR; CRISPR-mediated perturbation of *EZH2*, *DOT1L*, *KDM2A/3A*, or metabolic enzymes; rescue experiments; and functional assays in T cells or macrophages. This integrated framework can move the field from bulk-level correlation toward cell-type-specific and spatially resolved mechanisms while distinguishing observational associations, mechanistic evidence and therapeutic hypotheses.

## 5. Conclusions

In summary, histone methylation and tumor metabolic reprogramming are reciprocally connected. Metabolic intermediates, including SAM and α-KG, can influence the activity of histone-modifying enzymes, while histone methylation can alter the expression of genes involved in tumor metabolism and immune regulation. These interactions may affect tumor growth, metastatic behavior and antitumor immunity, but their effects vary across enzymes, genomic loci, cell types and metabolic states. The findings on tumor-associated macrophages and other immune cells should therefore be interpreted in the context of the experimental model rather than generalized using fixed polarization categories. Histone lactylation and other adjacent mechanisms may help explain specific links in this network but should be distinguished from histone methylation and included only where a direct connection is supported. Although these mechanisms suggest potential therapeutic opportunities, much of the evidence remains preclinical, and early clinical activity does not by itself establish improved outcomes. Future studies should define the cellular and metabolic contexts in which these interactions occur, test causality with appropriate experimental models, and evaluate biomarker-informed strategies in well-designed clinical trials.

From a clinical perspective, combined therapeutic strategies targeting histone methylation and metabolic pathways are being investigated in preclinical and early-phase studies. Clinical benefit remains to be established, and these strategies should not yet be described as overcoming drug resistance. Nevertheless, this field still faces numerous challenges, such as the incompletely understood molecular mechanisms of the synergistic effects between histone methylation and metabolic pathways, as well as the need to address drug resistance and avoid off-target effects. Future research should focus on dissecting the cross-regulatory networks between metabolism and histone methylation, leveraging cutting-edge technologies such as single-cell epigenomics and metabolomics to accurately capture the spatiotemporal dynamic characteristics of these interactions.

Ultimately, an in-depth understanding of the interaction between metabolic reprogramming and histone methylation is critical for developing precision medicine-based treatment regimens. Formulating personalized therapeutic interventions based on the unique epigenetic-metabolic profiles of individual tumors is expected to improve treatment efficacy and reduce adverse reactions. Sustained interdisciplinary collaboration and translational medical research are the key links driving the translation of relevant research from the laboratory to clinical applications, which will advance innovative therapies targeting the vulnerabilities of tumor metabolism and epigenetic regulation into clinical practice, bringing new breakthroughs to the field of cancer treatment.

## Figures and Tables

**Figure 1:**
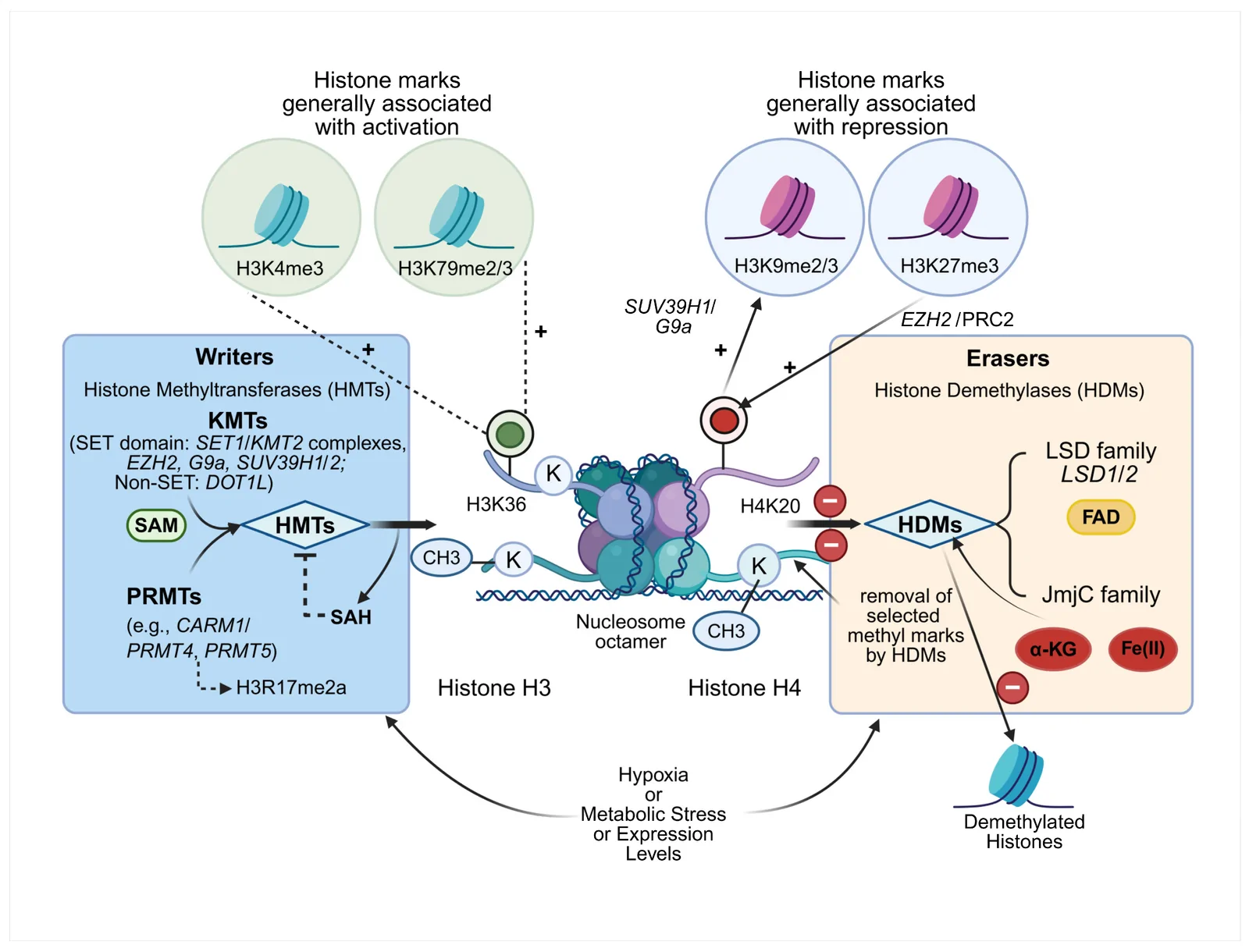
Histone-methylation writers, erasers and metabolic coupling (created with BioRender.com). Histone methyltransferases (HMTs) use S-adenosylmethionine (SAM) as the methyl donor to deposit methyl groups on specific histone residues; S-adenosylhomocysteine (SAH) is generated as a byproduct and can inhibit methyltransferase activity. Depending on the enzyme, residue and genomic context, these marks may be associated with transcriptional activation (e.g., H3K4me3, H3K79me2/3) or repression (e.g., H3K9me2/3, H3K27me3). *KMT2/COMPASS* complexes (including SET1A/SET1B) and *DOT1L* catalyze H3K4me3 and H3K79me2/3, respectively, whereas *SUV39H1* and *G9a/EHMT2* catalyze H3K9me2/3 and *EZH2*, the catalytic subunit of PRC2, catalyzes H3K27me3. Histone demethylases (HDMs) remove methyl groups through FAD-dependent *LSD 1/2* or Fe(II)/α-KG-dependent JmjC enzymes. Hypoxia, metabolite availability (e.g., SAM, α-KG, succinate, 2-HG) and enzyme expression levels can modulate this regulatory system in a context-dependent manner in tumors. Legend: Solid arrows indicate direct promotion; dashed arrows indicate indirect or potential regulation. "+" and "−" denote activation and inhibition, respectively. Blue box, Writers (HMTs); yellow box, Erasers (HDMs). Green circles, activation-associated marks (H3K4me3, H3K79me2/3); red circles, repression-associated marks (H3K9me2/3, H3K27me3). SAM, SAH, FAD, α-KG, and Fe(II) denote S-adenosylmethionine, S-adenosylhomocysteine, flavin adenine dinucleotide, α-ketoglutarate, and ferrous iron, respectively.

**Figure 2:**
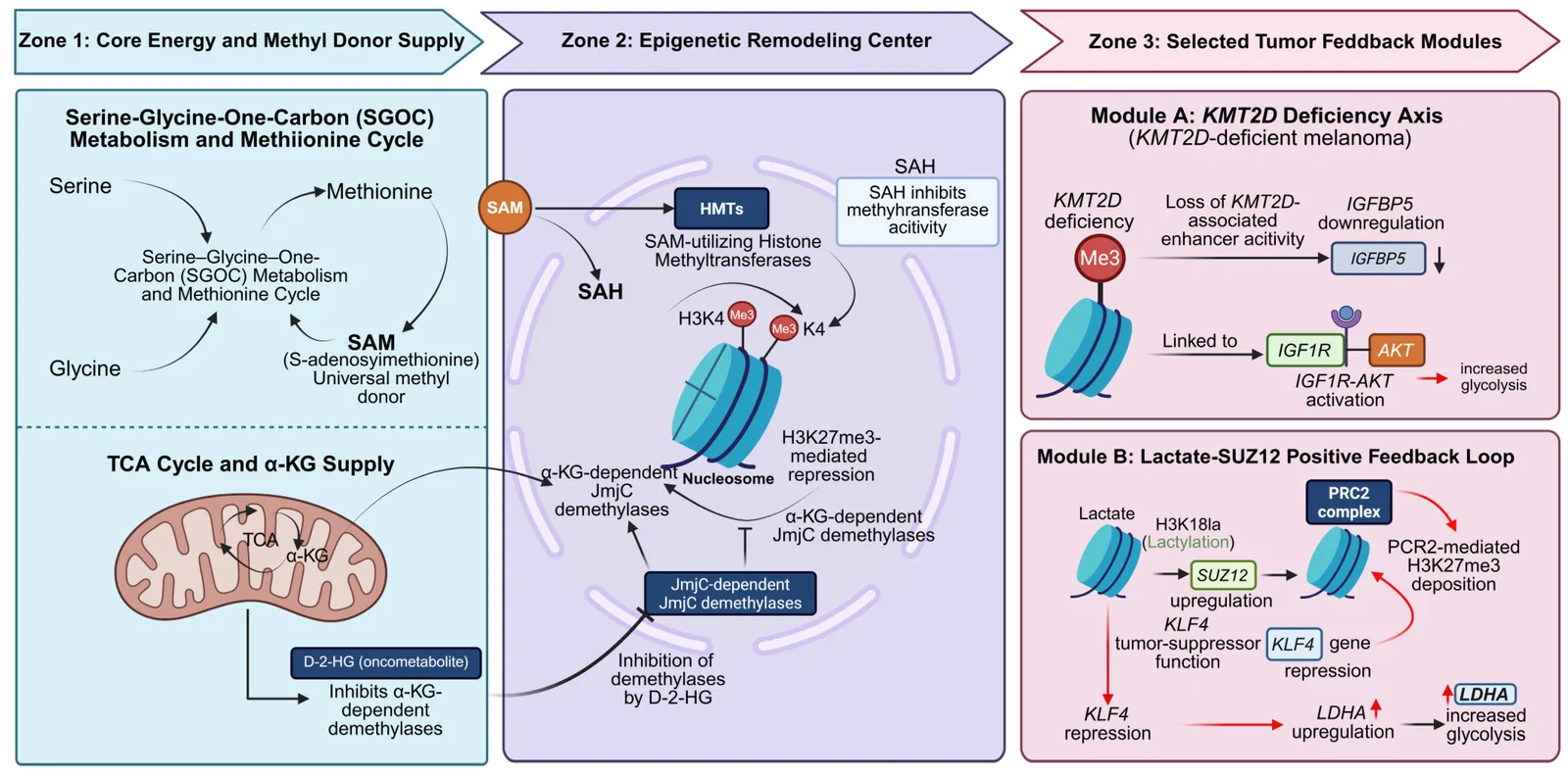
Selected tumor-specific metabolic–epigenetic feedback modules (created with BioRender.com). Zone 1 summarizes the serine–glycine–one-carbon (SGOC) metabolic, the methionine cycle and the TCA cycle as sources of SAM and α-KG. Zone 2 shows the chromatin interface, where histone methyltransferases use SAM (and generate SAH, which can inhibit methyltransferase activity) and JmjC-domain demethylases require α-KG and Fe(II); the oncometabolite D-2-HG, produced by IDH1/2-mutant tumors, can competitively inhibit α-KG-dependent demethylases. Zone 3 presents two selected tumor mechanisms. In reported melanoma models, loss of KMT2D-associated enhancer activity reduces IGFBP5 expression and is linked to IGF1R-AKT activation and increased glycolysis. In retinoblastoma models, lactate-associated H3K18 lactylation increases SUZ12 expression, enabling PRC2-mediated H3K27me3 deposition at the KLF4 locus, KLF4 repression and LDHA-dependent glycolysis. Histone lactylation is shown in Zone 3 (Module B). Symbols are defined in the figure key. Legend: Arrows (→) indicate promotion, flux, or positive regulation. Blunt-ended lines (⊣) indicate inhibition. Solid boxes represent pathways or modules; dashed boxes indicate selected tumor feedback modules. Colored zones (blue, purple, pink) represent Core Energy and Methyl Donor Supply, Epigenetic Remodeling Center, and Selected Tumor Feedback Modules, respectively. SAM, SAH, α-KG, D-2-HG, TCA, HMTs, and JmjC denote S-adenosylmethionine, S-adenosylhomocysteine, α-ketoglutarate, D-2-hydroxyglutarate, tricarboxylic acid, histone methyltransferases, and Jumonji C domain-containing demethylases, respectively.

**Figure 3:**
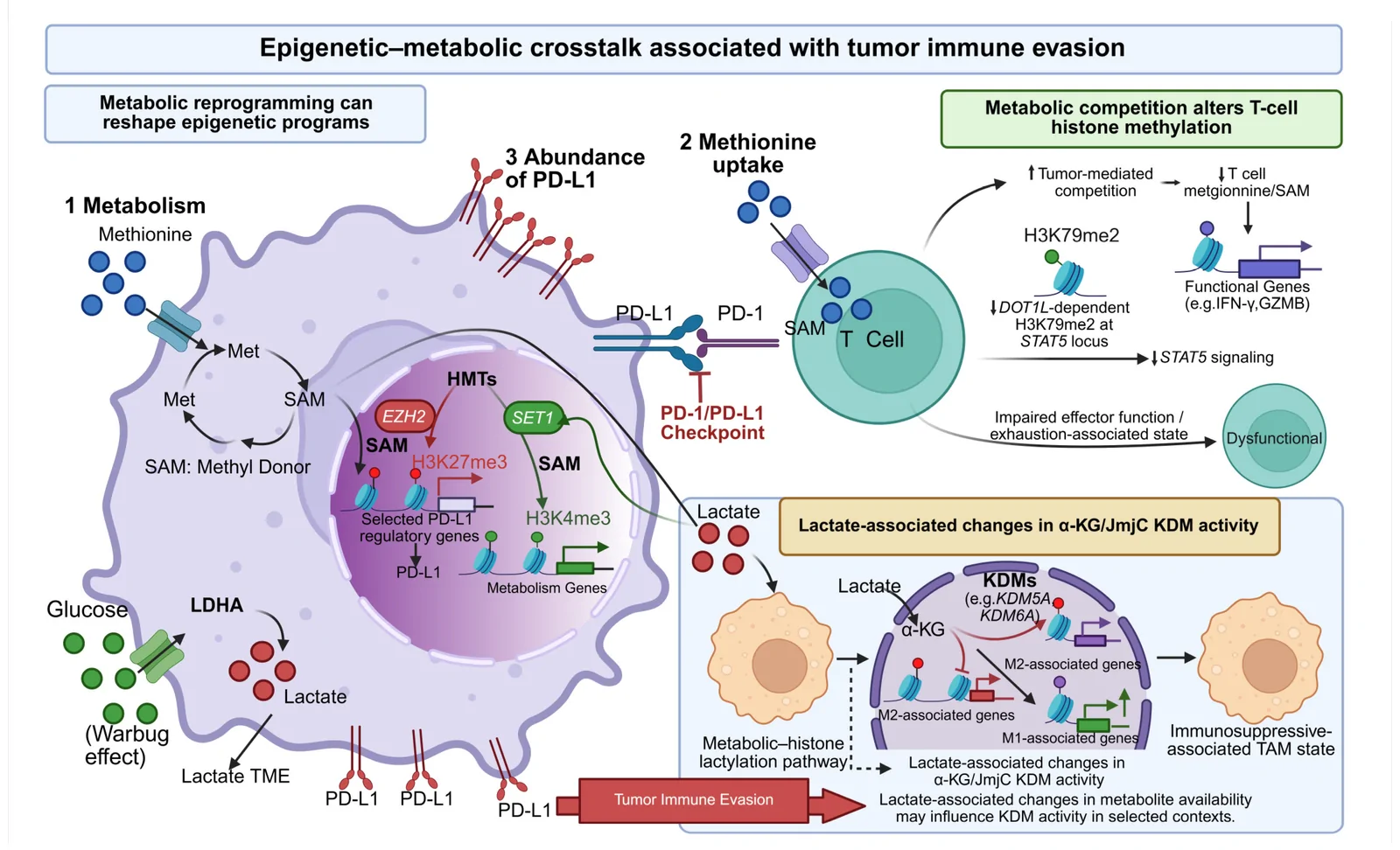
Selected metabolic-epigenetic pathways associated with tumor immune evasion (created with BioRender.com). In tumor cells, two independent epigenetic programs may influence selected regulators of *CD274*/PD-L1 expression: *EZH2*/PRC2-mediated H3K27me3 (repressive) and *SET1/KMT2*-associated H3K4me3 (activating); These effects are tumor- and context-dependent and should not be interpreted as a universal cooperative mechanism. Glycolysis-derived lactate contributes to an immunosuppressive tumor microenvironment and can induce histone lactylation in tumor-associated macrophages, which may upregulate PD-L1. In the methionine-competition model, *SLC43A2*-expressing tumor cells reduce methionine availability to neighboring CD8+ T cells, lowering intracellular SAM and *DOT1L*-dependent H3K79me2 at the *STAT5* locus, with reduced STAT5 signaling and impaired effector function. Any effect of lactate or other metabolites on α-KG-dependent demethylases (e.g., KDM5A, KDM6A) should be interpreted as context-dependent, as reported in selected models. The figure summarizes mechanisms reported in selected experimental systems and does not imply that all pathways converge directly on PD-1/PD-L1 signaling. Legend: Solid arrows (→) indicate direct promotion, metabolic flux, or positive regulation. Dashed arrows (⇢) indicate indirect, potential, or context-dependent regulation. Blunt-ended lines (⊣) indicate inhibition. The blue box represents metabolic reprogramming; the green box represents T-cell histone methylation; the orange box represents lactate-associated changes in α-KG/JmjC KDM activity. SAM, SAH, α-KG, TME, HMTs, KDMs, and PD-L1 denote S-adenosylmethionine, S-adenosylhomocysteine, α-ketoglutarate, tumor microenvironment, histone methyltransferases, histone demethylases, and programmed death-ligand 1, respectively.

**Figure 4:**
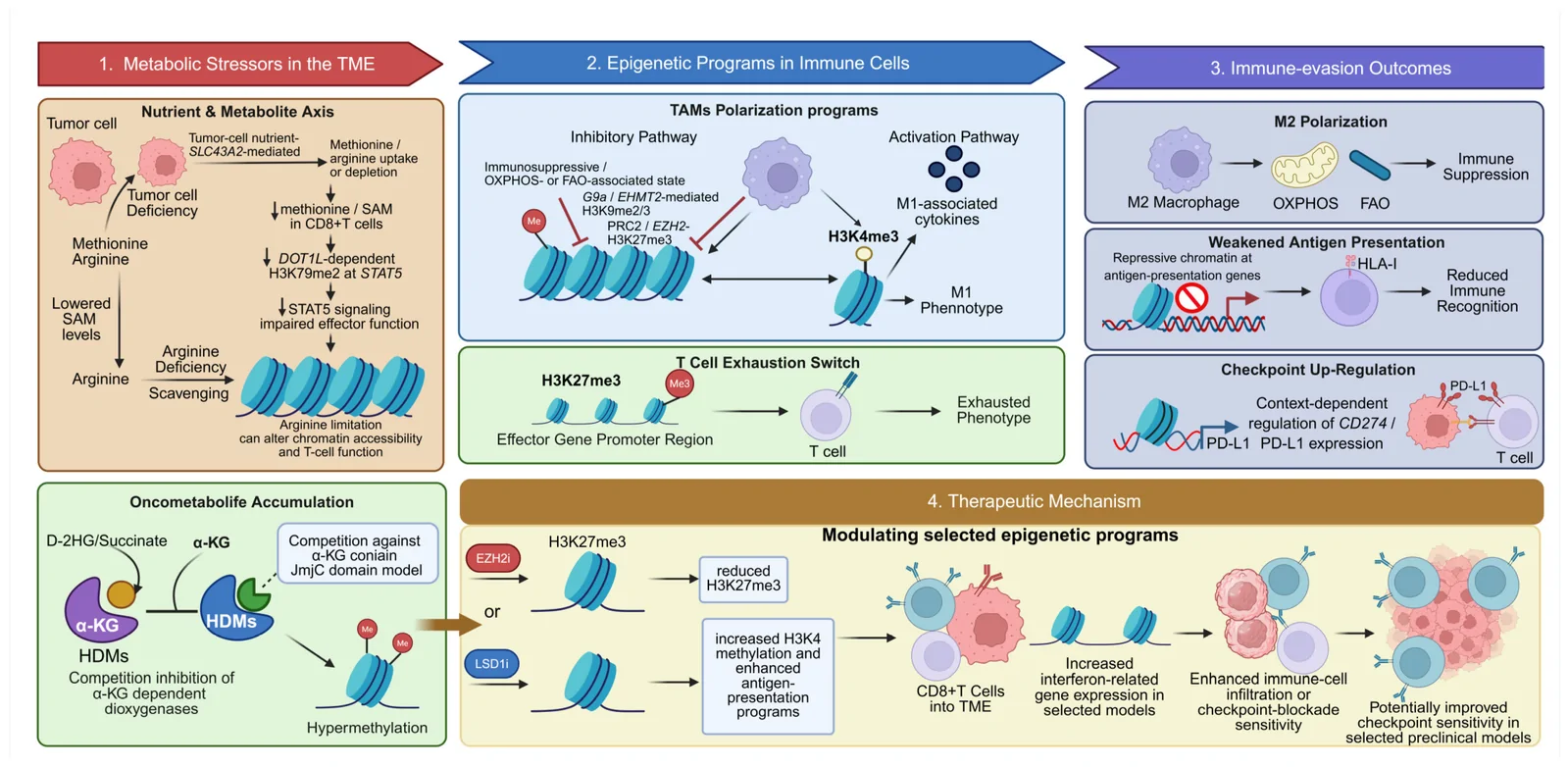
Context-dependent metabolic and epigenetic mechanisms associated with immune evasion and therapeutic response in the tumor microenvironment (created with BioRender.com). Tumor-cell or myeloid-derived suppressor cell consumption of methionine and arginine can reduce nutrient availability to infiltrating T cells. In the *SLC43A2* model, competition by *SLC43A2*-expressing tumor cells lowers SAM and reduces *DOT1L*-dependent H3K79me2 at the *STAT5* locus, whereas arginine limitation can alter chromatin accessibility and T-cell function. D-2HG (from *IDH1/2*-mutant tumors) and succinate (from *SDH*-mutant tumors) can inhibit α-KG-dependent dioxygenases, but the resulting methylation changes depend on metabolite concentration, mutation status and cellular compartment. TAMs are heterogeneous and plastic. *G9a/EHMT2-*, *EZH2-*, and H3K4me3-associated programs may bias macrophages toward inflammatory/glycolytic, immunosuppressive/OXPHOS-, or FAO-associated, or intermediate states; these states do not map onto a fixed M1/M2 dichotomy. H3K27me3 at selected effector-gene loci has been associated with exhaustion-related transcription in particular T-cell contexts in selected models. Altered antigen presentation and context-dependent regulation of *CD274*/PD-L1 may contribute to immune escape. Inhibition of *EZH2* or *LSD1* can modulate selected epigenetic programs and has increased interferon-related gene expression, immune-cell infiltration or checkpoint-blockade sensitivity in selected preclinical models; clinical benefit remains to be established. Legend: Solid arrows (→) indicate direct promotion, metabolic flux, or positive regulation. Dashed arrows (⇢) indicate indirect, potential, or context-dependent regulation. Blunt-ended lines (⊣) indicate inhibition. The red box represents metabolic stressors in the TME; the blue box represents epigenetic programs in immune cells; the purple box represents immune-evasion outcomes; the brown box represents therapeutic mechanisms. α-KG, D-2-HG, TME, TAMs, OXPHOS, FAO, HDMs, KDMs, and PD-L1 denote α-ketoglutarate, D-2-hydroxyglutarate, tumor microenvironment, tumor-associated macrophages, oxidative phosphorylation, fatty acid oxidation, histone demethylases, histone demethylases, and programmed death-ligand 1, respectively.

**Table 1:** Selected histone methyltransferases and demethylases implicated in cancer.

Subclass	Modified Residue	HMTs and HDMs Involved in Tumor Diseases	Current Gene Symbol	Types of Tumor Diseases	References
Lysine methyltransferases (KMTs)	-	-	-	-	-
KMT1	H3K9	*SUV39H1*	KMT1A	HBV-associated hepatocellular carcinoma, melanoma; lung cancer; breast cancer; leukemia	[29,30,31]
		*G9a(EHMT2)*	KMT1C	Lung cancer, breast cancer, leukemia	[32,33]
KMT2	H3K4	*MLL2*	KMT2D	Melanoma (enhancer remodeling, glycolysis); other tumor associations are model- or review-level	[34,35]
		*MLL3*	KMT2C	Breast cancer, colorectal cancer	[35,36]
KMT3	H3K4	*SMYD2*	KMT3C	Prostate cancer, Bladder cancer	[37]
	H3K36	*SMYD3*	KMT3E	Breast cancer, Hepatocellular carcinoma	[38]
		*NSD2*	KMT3G	Multiple myeloma, triple-negative breast cancer	[39]
KMT5	H4K20	*SETD8*	KMT5A	Neuroblastoma, lung cancer	[40,41]
		*SUV4-20H*	KMT5B-C	prostate cancer, pancreatic cancer	[42,43]
KMT6	H3K27	*EZH2*	KMT6A	Colorectal cancer	[44]
Lysine demethylases (KDMs)	-	-	-	-	-
KDM1	H3K4/H3K9	*LSD1 (KDM1A)*	KDM1A	Small cell lung cancer, breast cancer, squamous cell carcinoma	[45,46]
KDM2	H3K36	*KDM2A*	KDM2A	Head and neck squamous cell carcinoma	[47]
KDM3	H3K9	*JMJD1A (KDM3A)*	KDM3A	kidney cancer	[48]
		*JMJD1C (KDM3C)*	KDM3C	AML, Prostate cancer, esophageal cancer, colorectal cancer	[49,50]
KDM4	H3K9	*JMJD2A (KDM4A)*	KDM4A	Prostate cancer, bladder cancer	[51]
KDM5	H3K4	*KDM5A (JARID1A)*	KDM5A	Breast cancer, pancreatic cancer	[52,53]
KDM6	H3K27	*UTX (KDM6A)*	KDM6A	Bladder cancer, kidney cancer	[54,55]
		*JMJD3 (KDM6B)*	KDM6B	Non-small cell lung cancer, colon cancer, breast cancer	[56,57]
Protein arginine methyltransferases (PRMTs)	-	-	-	-	-
Type I PRMT	H3R17	*PRMT4 (CARM1)*	CARM1	Liver cancer, breast cancer, prostate cancer	[58]
Type II PRMT	H3R8me2s/H4R3me2s (symmetric)	*PRMT5*	PRMT5	Pancreatic cancer, prostate cancer, lung cancer	[59,60]

**Table 2:** Key metabolites at the histone methylation–chromatin interface.

Metabolite	Source Pathway	Target (Enzyme/Modification)	Regulatory Effect	References
S-Adenosylmethionine (SAM)	Methionine cycle/One-carbon metabolism	HMTs (methyl donor; context-dependent)	Methyl donor for activating and repressive marks; effect depends on enzyme, residue and locus. Reduced SAM in CD8+ T cells lowered DOT1L-dependent H3K79me2 at STAT5 in the SLC43A2 model.	[88,97]
Alpha-Ketoglutarate (α-KG)	Tricarboxylic acid cycle (TCA)	JmjC family HDMs (cofactor)	Required cofactor for JmjC demethylases; activity depends on the specific demethylase, residue, locus and the α-KG/succinate ratio.	[98,99]
2-Hydroxyglutaric acid (2-HG)	IDH-mutant tumors	JmjC family HDMs (inhibitor)	Competitive inhibition of α-KG-dependent demethylases; drives histone hypermethylation in IDH-mutant AML and glioma.	[9,11]
S-adenosylhomocysteine (SAH)	Methionine cycle/transmethylation	may reduce methyltransferase activity depending on the SAM/SAH ratio	Elevated SAH and a reduced SAM/SAH ratio inhibit methyltransferase reactions and alter H3K9 methylation in hepatoma cells.	[100]

**Table 3:** Selected context-dependent links between histone methylation and immune-cell metabolism or function.

Types of Immune Cells	Key Methylation Modifications	Regulation of Enzymes	Metabolic/ Epigenetic Changes	Functional Impact (Tumor-Related)	References
Tumor-associated macrophages (TAMs)	H3K27me3	EZH2	A polarization state that the authors described as M2-like in an HCC model; not a universal TAM program	Context-dependent role for EZH2 in TAM-state regulation; not a universal M1/M2 program	[125]
CD8+ T cells	H3K79me2	DOT1L	Methionine competition reduced SAM and H3K79me2 at STAT5 in the SLC43A2 model	Impaired effector function in the reported model; DOT1L effects vary with T-cell state	[88,110]
	H3K9me3	CD8+ T cell-intrinsic SUV39H1	SUV39H1-mediated H3K9me3 in a melanoma model	Associated with anti-PD-1 responsiveness; model-specific	[31]
Dendritic cells (DCs)	H3K79me2	DOT1L	L-lysine altered DC metabolism and histone methylation	Associated with a tolerogenic phenotype in the cited system.	[109]
	H3K4me3/H3K27me3	β2-integrin-regulated (enzyme not specified in the cited study)	Altered chromatin accessibility during DC maturation	β2-integrin restricts DC maturation and tumor rejection in the cited model	[126]

**Table 4:** Preclinical and Clinical Research Progress of Histone Methylation Inhibitors.

Targeted Enzyme	Inhibitor	Development or Regulatory Status	Tumor Type	Core Mechanism of Action	References
EZH2	Tazemetostat	FDA-approved for R/R FL with EZH2 mutation (Y646, A682, A692) and for R/R FL without satisfactory alternative options (accelerated approval)	R/R follicular lymphoma (EZH2-mutant and wild-type cohorts); other EZH2-dependent malignancies under investigation	EZH2 inhibition reduces H3K27me3; downstream effects depend on tumor genotype and context	[133,134,135,136]
	GSK2816126	Phase I	Advanced hematologic and solid tumors	Phase I study reported limited activity; the cited CML study used a different EZH2 inhibitor, GSK126, and represents a separate preclinical context	[137,138]
DOT1L	Pinometostat (EPZ-5676)	Phase I	KMT2A-rearranged leukemia, formerly termed MLL-rearranged leukemia	Inhibits DOT1L-mediated H3K79 methylation; A phase I study in acute leukemia reported modest clinical activity	[67,127,139]
G9a (EHMT2)	BIX01294	Preclinical	lung adenocarcinoma, pancreatic ductal adenocarcinoma	Inhibits H3K9me2, induce ROS generation	[140,141]
LSD1	Iadademstat (ORY-1001)	Phase I/II	Acute myeloid leukemia, pancreatic cancer	LSD1/KDM1A inhibitor; blocks LSD1-mediated H3K4 demethylation and induces differentiation in AML	[142,143,144]
PRMT5	PF-06939999/AMG 193/MRTX1719	Phase I/II	MTAP-deleted solid tumors	MTA-cooperative inhibition, synthetic lethality in MTAP-deleted cancers	[145,146,147]

**Table 5:** Selected clinical studies of epigenetic inhibitors targeting histone methyltransferases and demethylases in hematological malignancies and solid tumors.

Clinical Trial Identifier	Drug(s) Tested	Disease Tested	Biomarker	Results
NCT04102150 (Phase II)	Valemetostat	R/R ATL	*EZH1/2*	ORR 48.0% (5 CR, 7 PR); durable response; manageable safety [96].
NCT02082977 (Phase I)	GSK2816126	R/R NHL and solid tumors	*EZH2* inhibitor	1 PR (lymphoma), 34% SD; MTD 2400 mg; all patients experienced AEs, 32% serious AEs; modest anticancer activity [137].
NCT01897571 (Phase II)	Tazemetostat (withdrawn from global market in 2026 due to secondary hematologic malignancy risk)	R/R FL	*EZH2*	ORR 69% (mutated cohort); durable response (mPFS 13.8 mo); manageable safety [136].
NCT02860286 (Phase II)	Tazemetostat (historical data)	R/R MPM	*BAP1*	12-week DCR 54% (PR only); manageable toxicity [162].
NCT03854227 (Phase I)	PF-06939999	Advanced solid tumors	*PRMT5*	3 PR, 19 SD; manageable hematological toxicity [145].
NCT05094336 (Phase I/II)	AMG193	MTAP-deleted solid tumors	*MTAP, PRMT5*	ORR 21.4% (95% CI 10.3–36.8); manageable safety [146].
NCT05245500 (Phase I/II)	MRTX1719	MTAP-deleted solid tumors	*MTAP, PRMT5*	Objective responses in MTAP-deleted tumors; ORR not reported [147].
NCT01684150 (Phase I)	Pinometostat (EPZ-5676)	MLL-rearranged leukemia	*DOT1L*, H3K79	2 CR (t(11;19)+); modest clinical activity; manageable safety [67].
NCT02034123 (Phase I)	GSK2879552	R/R SCLC	*LSD1*	Limited clinical activity; poor tolerability (83% TRAEs); significant safety concerns [163].
NCT02261779 (Phase I/II)	TCP	R/R AML	*LSD1*, H3K4	ORR 20% (2 CRi, 1 PR); mOS 3.3 months; manageable safety [164].

## Data Availability

No new data were created or analyzed in this study. Data sharing is not applicable to this article.

## Abbreviations

HMTs	Histone Methyltransferases
HDMs	Histone Demethylases
KMTs	Histone Lysine Methyltransferases
PRMTs	Protein Arginine Methyltransferases
SAM	S-adenosylmethionine
SAH	S-adenosylhomocysteine
α-KG	α-ketoglutarate
2-HG	2-hydroxyglutarate
PTM	Post-translational Modification
TME	Tumor Microenvironment
TAMs	Tumor-associated Macrophages
CAFs	Cancer-associated Fibroblasts
EMT	Epithelial-mesenchymal Transition
TCA cycle	Tricarboxylic Acid cycle
OXPHOS	Oxidative Phosphorylation
ICB/ICIs	Immune Checkpoint Blockade/Inhibitors
ROS	Reactive Oxygen Species
TSSs	Transcription Start Sites
SGOC	Serine-glycine-one-carbon
TCP	Tranylcypromine
